# Function of serine/arginine-rich splicing factors in hematopoiesis and hematopoietic malignancies

**DOI:** 10.1186/s12935-024-03438-7

**Published:** 2024-07-21

**Authors:** Huifang Zhang, Hongkai Zhu, Hongling Peng, Yue Sheng

**Affiliations:** 1 Department of Hematology, the Second Xiangya Hospital, Central South University, Changsha, 410011 Hunan P. R. China; 2 Hunan Engineering Research Center of Targeted therapy for Hematopoietic Malignancies, Changsha, 410011 Hunan P. R. China

**Keywords:** Serine/arginine-rich splicing factors, Hematopoiesis, Hematopoietic malignancies

## Abstract

The serine/arginine-rich splicing factors (SRSFs) play an important role in regulating the alternative splicing of precursor RNA (pre-RNA). During this procedure, introns are removed from the pre-RNA, while the exons are accurately joined together to produce mature mRNA. In addition, SRSFs also involved in DNA replication and transcription, mRNA stability and nuclear export, and protein translation. It is reported that SRSFs participate in hematopoiesis, development, and other important biological process. They are also associated with the development of several diseases, particularly cancers. While the basic physiological functions and the important roles of SRSFs in solid cancer have been extensively reviewed, a comprehensive summary of their significant functions in normal hematopoiesis and hematopoietic malignancies is currently absent. Hence, this review presents a summary of their roles in normal hematopoiesis and hematopoietic malignancies.

**Graphical Abstract:**

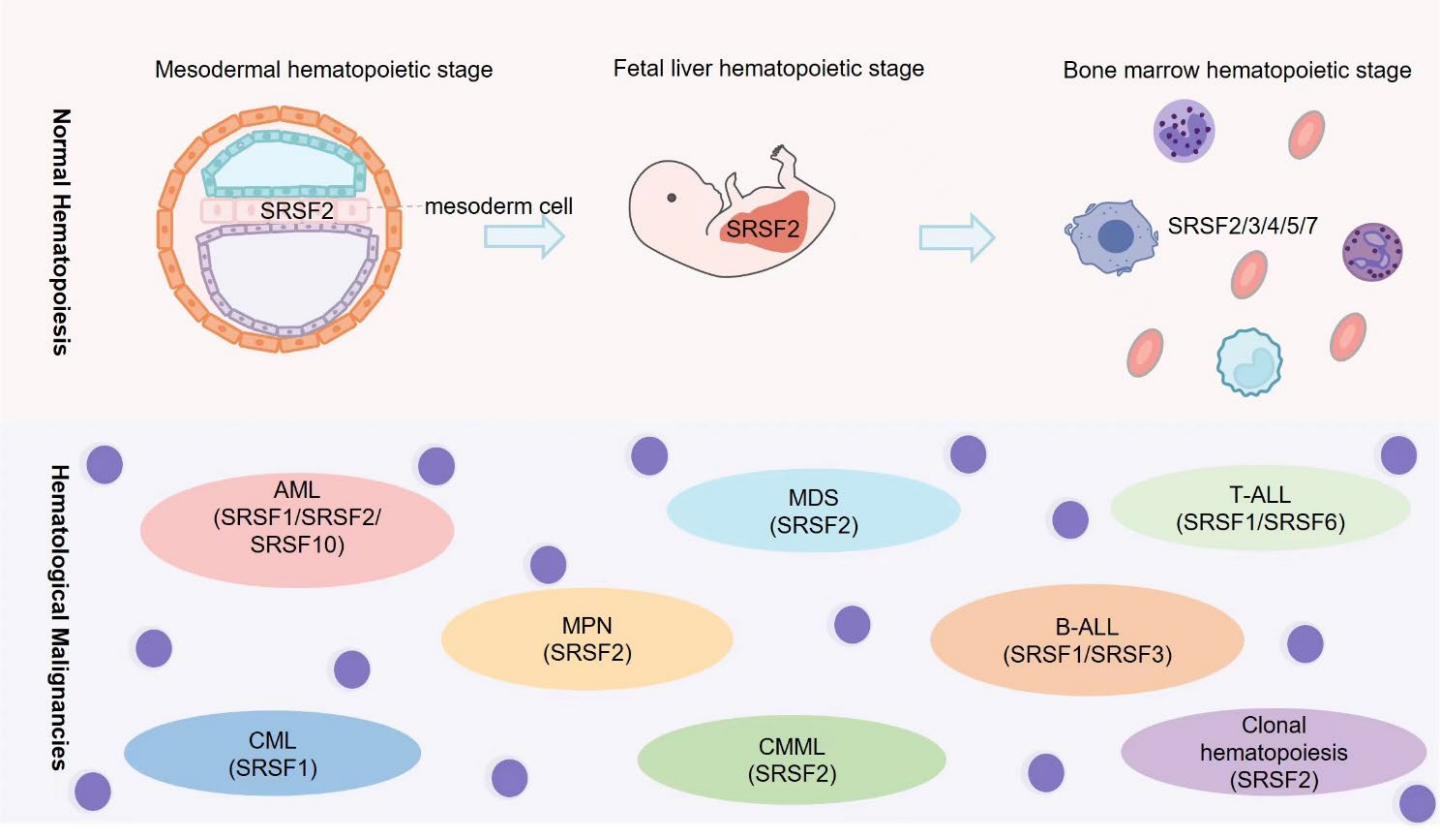

## Introduction

Gene expression is a complex process involving various steps, including transcription, translation, post-transcription and post-translation. Among these steps, the splicing of precursor RNA (pre-RNA) was important in the post-transcription step. In the process of splicing, the introns are removed from the pre-RNA, while the exons are accurately joined together to produce mature mRNA. The spliceosome complex, composed mainly of five small nuclear ribonucleoprotein particles (snRNPs), aids in the splicing process of pre-RNA by recognizing and splicing it [1].

The process of splicing is highly controlled and regulated by several components referred to as splicing regulatory elements (SREs), including cis-acting factors and trans-acting factors [1]. Cis-acting factors include exonic splicing enhancers (ESEs), exonic splicing silencers (ESSs), intronic splicing enhancers (ISEs), and intronic splicing silencers (ISSs), which are located within the pre-RNA sequence [2]. Trans-acting factors can bind to cis-acting factors and regulate spliceosome assembly and splice site recognition. The serine/arginine-rich splicing factors (SRSFs), consisting of 12 members (SRSF1-SRSF12) in mammals, were reported to be important trans-acting factors. SRSFs primarily bind to the cis-acting element ESEs and facilitate the binding of snRNPs to the splicing sites, thereby regulating pre-RNA splicing [3].

Apart from their role in pre-RNA splicing, SRSF family members also play important roles in DNA replication, transcription, and translation, thereby involves in hematopoiesis, development, and other biological process [4–7]. They are also associated with the development of several diseases, particularly cancers. While the physiological functions of SRSFs and their involvement in solid cancer development have been extensively reviewed [8–12], a comprehensive summary of their significant functions in normal hematopoiesis and hematopoietic malignancies is currently absent. Hence, the review seeks to present a summary of their roles in normal hematopoiesis and hematopoietic malignancies.

## Structures of SRSF family members

With the exception of SRSF12, which is predominantly expressed in the brain and testis, the other members of the SRSF families exhibit wide expression across various tissues [13]. While most SRSFs are localized exclusively in the nucleus, certain members, such as SRSF1, SRSF3, and SRSF7, have the ability to shuttle between the nucleus and cytoplasm [10].

SRSF proteins exhibit a remarkably conserved structure, usually comprising one or two RNA recognition domains (RRMs) positioned at the N-terminal region. At the carboxyl terminus, there is a highly phosphorylated serine/arginine-rich domain (RS) [10]. The RRM domain is responsible for recognizing specific RNA sequences and determining the binding sites of SRSFs on RNA. The RS domain facilitates interactions between SRSFs and other proteins as well as RNA, and its activity can be regulated through phosphorylation modifications [12].

## Molecular functions of SRSFs

### SRSFs are associated with alternative splicing of pre-RNA

**Fig. 1:**
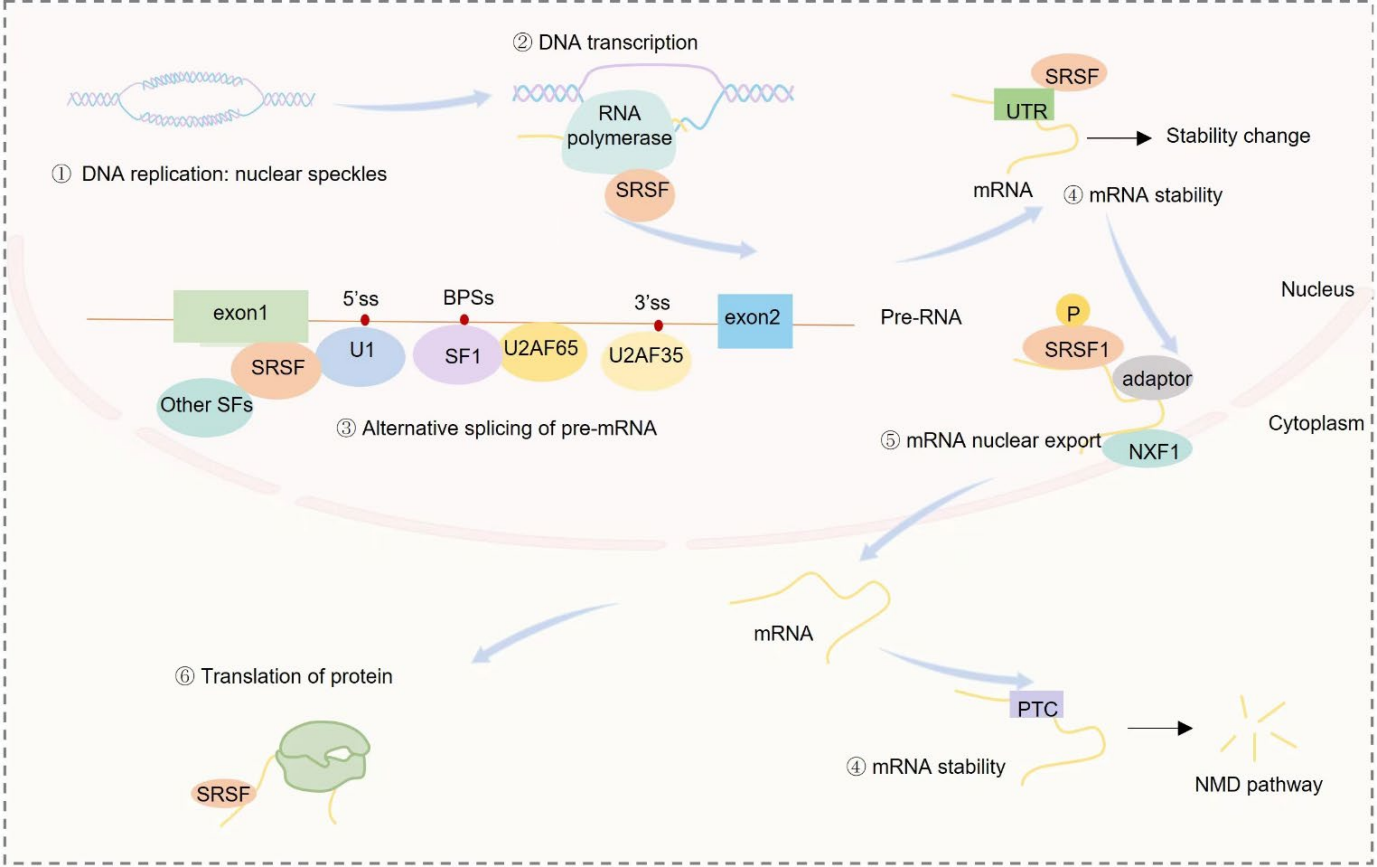
The molecular functions of SRSFs. ① SRSF proteins are primarily located in nuclear speckles, where DNA replicate and transcript. ② SRSFs can interact with the RNA polymerase II and involve in transcription. ③ Splice sites include 5’ splice sites (5’ss), 3’ splice sites (3’ss), and branch point sites (BPSs), which can be recognized and spliced by small nuclear ribonucleoproteins (snRNPs) (U1, U2, U4, U5, and U6). The splicing process begins with U1 snRNP recognizing the 5’ss, simultaneously, U2AF65 interacts with splicing factor 1 (SF1) to facilitate its binding to the BPSs. Additionally, U2AF35 binds to the 3’ss. SRSFs can act as a bridge between pre-mRNA and U1 snRNP. In addition, SRSFs can also interact with other proteins and recognize specific sequences within pre-mRNA. ④ SRSFs can directly bind to mRNA and affect its stability; in addition, alternative splicing events mediated by SRSFs can introduce a premature termination codon (PTC) in the mRNA transcript, resulting in decreased mRNA stability and degradation through the nonsense-mediated mRNA decay (NMD) pathway in the cytoplasm. ⑤ Reduced binding of phosphorylated SRSF1 to target mRNA promotes the binding of RNA to the nuclear export adaptor ALYREF and the nuclear export receptor Nxf1, facilitating the export of mRNA from the nucleus to the cytoplasm. ⑥ SRSFs can inhibit the translation binding to the UTR region of specific target

SRSFs play crucial roles in alternative splicing of pre-mRNA [9]. During gene expression, pre-mRNA, which contains multiple introns, is processed by the spliceosome complex to generate mature RNA and produce different transcript variants. Splice sites can be categorized into three types: 5’ splice sites (5’ss), 3’ splice sites (3’ss), and branch point sites (BPSs) located 18–40 nucleotides upstream of the 3’ss [15]. Additionally, there is a polypyrimidine region following the BPSs. The spliceosome, assembled by five types of small nuclear ribonucleoproteins (snRNPs) (U1, U2, U4, U5, and U6), can recognize the splice sites and cleave the RNA chain to remove introns [16, 17] (Fig. 1).

The splicing process begins with U1 snRNP recognizing the 5’ss through complementary base pairing [18]. Simultaneously, U2AF65 binds to the polypyrimidine region and attaches to splicing factor 1 (SF1) to facilitate its binding to the BPSs. Additionally, U2AF35 attaches to the 3’ss, resulting in the formation of the E complex. Subsequently, with the assistance of U2AF, U2 snRNP takes the place of SF1 and attaches to the BPSs through base complementary matching, resulting in the formation of complex A. The triple snRNP particles (U4, U6, and U5) induce rearrangement of complex A, bringing the three splice sites together. By undergoing a series of structural changes, U1 snRNP separates and U6 snRNP attaches to the 5’ss, enabling the formation of a base-paired complex between U6 snRNP and U2 snRNP. Following this rearrangement, complex B, which possesses catalytic activity, is formed and can splice the pre-mRNA through two transesterification reactions [18, 19].

SRSF proteins facilitate splice site recognition, mediate spliceosome assembly, and participate in alternative splicing [20]. Alternative splicing events regulated by SRSFs include alternative 5’ss, alternative 3’ss, skipped exons, retained introns, and mutually exclusive exons [21]. SRSFs possess distinct binding motifs within pre-mRNA. For example, SRSF1 preferentially binds to the consensus sequence GGAGA within exonic regions [22]. The binding motifs of SRSF3 are mainly CCAGC(G)C and A(G)CAGCA, while SRSF9 binds to pre-mRNA motifs rich in GA, typically located in the coding sequence (CDS) region and 5’ untranslated region (UTR) of the coding exon [23]. Notably, the proportion of bound RNA molecules in pre-mRNA is correlated with the number of cis-acting elements [24].

Moreover, SRSFs assist in spliceosome assembly. For example, the RNA recognition motifs (RRMs) of SRSF1 act as a bridge between pre-mRNA and the RRM of the U1-70 K component of U1 snRNP [25] (Fig. 1). SRSFs can also interact with other proteins and recognize specific sequences within pre-mRNA, leading to changes in signal intensity of splicing at splice sites. SRSFs can compete or cooperate in a dose-dependent manner to regulate alternative splicing. For instance, as negative regulators of poison exon inclusion in TRA2β, SRSF1 and SRSF3 synergistically increase protein levels of TRA2β by a log_2_5-fold, which is greater than the effect of either protein alone, indicating a synergistic effect [26].

### SRSFs regulate mRNA stability and nuclear export

SRSF proteins can influence mRNA stability through various mechanisms. Firstly, SRSFs can directly bind to mRNA and affect its stability (Fig. 1). For instance, SRSF2 and SRSF3 are primarily involved in mRNA stabilization, whereas SRSF1 facilitates mRNA degradation through its interaction with the 3’ untranslated region (3’UTR) of mRNA [27]. Secondly, alternative splicing can lead to the formation of a premature termination codon (PTC) in the mRNA transcript, which can cause a reduction in mRNA stability and degradation via the nonsense-mediated mRNA decay (NMD) pathway [28, 26] (Fig. 1). SRSF3 stabilizes the mRNA of TAR DNA-binding protein-43 (TDP43) by inhibiting the NMD pathway in triple-negative breast cancer, thereby maintaining the stemness of breast cancer stem cells [28]. Deletion of SRSF3 expression leads to the generation of a PTC in *TDP43* mRNA during alternative splicing, resulting in consistent mRNA levels in the nucleus and subsequent degradation of mRNA in the cytoplasm via the NMD pathway [29].

Furthermore, SRSFs are involved in the regulation of mRNA nuclear export. Reduced binding of phosphorylated SRSF1 to target mRNA enhances the attachment of RNA to the nuclear export adaptor ALYREF and the nuclear export receptor Nxf1, facilitating the transportation of mRNA from nucleus to cytoplasm [30] (Fig. 1). Additionally, SRSF3 can interact with the nuclear m^6^A reader YTHDC1 to mediate the nuclear export of N6-methylated mRNA [31].

### SRSFs maintain DNA replication and transcription

SRSF proteins are primarily located in nuclear speckles, which are substructures within the nucleus that lack a membrane. During adenovirus infection, SRSF2 assists in viral DNA replication and transcription within the nuclear speckle region [6] (Fig. 1). Additionally, SRSFs have the ability to interact with the carboxyl terminus of RNA polymerase II, suggesting their involvement in transcription (Fig. 1). Further studies have shown that SRSF2 can enhance the dynamic binding of positive transcription elongation factor b (P-TEFb) to RNA polymerase II, thereby facilitating phosphorylation of the carboxyl terminus of RNA polymerase II and promoting transcriptional elongation [32].

### SRSFs are involved in protein translation

SRSFs proteins are involved in the regulation of protein translation through direct binding to target mRNA and modulation of the activity of translation repressor 4E-BP [33, 34]. SRSF1 inhibits the translation of PTEN by suppressing the activity of the PTEN mRNA’s 3’-UTR^34^ (Fig. 1). Similarly, SRSF3 binds to the 5’-UTR region of specific target mRNAs, leading to the inhibition of translation of programmed cell death 4 (PDCD4) mRNA [35]. Moreover, SRSF1 can promote the translation of target mRNAs by regulating phosphorylation of the translation repressor 4E-BP, thus activating the translation-initiation factor eIF4E [33].

## The regulatory mechanisms of SRSFs

The expression and functions of SRSF family members are tightly regulated at both the transcriptional and posttranscriptional levels. First, SRSF family members can regulate each other’s transcription and alternative splicing. For example, SRSF1 can control the transcription of SRSF3, while SRSF3 can influence the alternative splicing of *SRSF1* pre-mRNA [36]. Second, the m^6^A modification level of *SRSF* mRNA can affect its stability and degradation through the YTHDC1-dependent NMD pathway, leading to changes in SRSF protein expression [37, 38]. Third, microRNAs can regulate SRSF1 expression by modulating the activity of mRNA’s 3’-UTR, thereby controlling its translation and protein levels [35]. Forth, SRSF2 can interact with its own mRNA through a disordered amino acid sequence at its carboxyl terminus, leading to the inhibition of its own translation [39]. In addition, SRSF3 controls its own expression by increasing the incorporation of a different exon 4 containing a stop codon [40]. Fifth, arginine methylases can modulate the methylation level of SRSF proteins, affecting their affinity for target mRNA and subsequent regulatory functions [41]. Sixth, various protein kinases, including AMPK, CDC like kinase (CLK), and SR protein kinases (SRPK), can regulate the activity and nucleoplasmic distribution of SRSF proteins through phosphorylation [42–44]. Seventh, protein hydroxylases and deubiquitination enzyme USP7 can impact the stability of SRSF proteins, influencing their abundance and activity [45, 46]. All these regulatory mechanisms ensure the precise control of SRSF expression and functions, allowing for dynamic regulation of RNA processing and gene expression.

## Important roles of SRSFs in development

SRSFs play crucial roles in promoting embryonic development and maintaining physiological functions. Srsf3 exhibits high expression levels in oocytes and during early embryonic development. Its deletion can result in the failure of blastocyst formation and embryonic death during the morula stage [21]. Mice lacking *Srsf3* in embryonic hearts display impaired cardiomyocyte proliferation and perish in utero [27, 47]. Srsf3 is also essential for the maturation and metabolic function of mouse livers [48]. Specific deletion of *Srsf3* in the liver impairs hepatocyte maturation and metabolism, leading to spontaneous hepatocellular carcinoma in mice [49].

SRSF2 promotes epidermal differentiation by regulating m^5^C modification of *VTRNA1.1* mRNA. Deletion of SRSF2 in epidermal progenitors can cause cell cycle arrest and cell death [50]. SRSF1 directly regulates the alternative splicing of *IRF7* and *IL27ra* mRNA, ensuring T cell maturation in the thymus and promoting thymus development [4]. T cell-specific *Srsf1* null mice can develop systemic autoimmune diseases and lupus nephritis due to excessive T cell activation and production of proinflammatory cytokines [35].

Multiple SRSFs are involved in the alternative splicing of the proinflammatory molecule Tissue Factor (TF). SRSF1 and SRSF6 promote exon 5 inclusion, while SRSF2 and SRSF5 promote exon 5 skipping through their competition for specific binding sites on exon 5 of *TF* mRNA [51]. Additionally, SRSF9 plays a major role as an alternative splicing factor in neutrophils [52].

## SRSFs promote tumorigenesis and progression

**Fig. 2:**
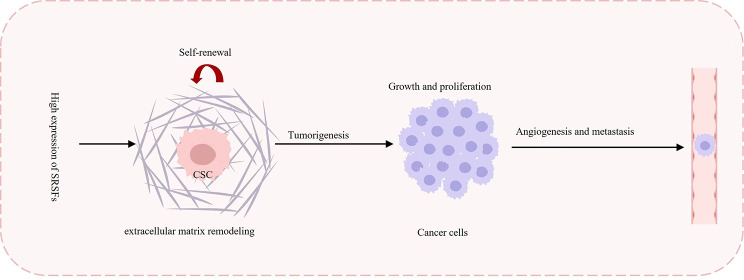
Roles of SRSFs in solid tumors. High expression of SRSFs in solid tumors can promote the growth and self-renewal of cancer stem cells (CSCs), maintain the growth and survival of cancer cells, induce extracellular matrix remodeling, cause angiogenesis and metastasis

High expression of SRSFs was associated with poor prognosis in various types of tumors, including ovarian cancer, gastric cancer, colorectal cancer, breast cancer, hepatocellular carcinoma, lung cancer, and glioma [53, 54]. First, SRSFs are essential for the growth and survival of cancer cells [53, 54]. In gastric cancer, SRSF1 directly binds to *MST1R* mRNA, regulating its splicing and promoting malignant tumor cell proliferation [55]. Second, in triple-negative breast cancer, SRSF3 interacts with the splicing factor TDP43 to regulate various splicing events, including apoptosis, extracellular matrix remodeling, cell adhesion, and tumor cell metastasis [56]. Third, SRSFs can also modulate the splicing of *VEGF* and *fibronectin* mRNA, promoting tumor angiogenesis and breast cancer progression in the tumor microenvironment [57]. Fourth, SRSF3 directly binds to *CCDC50S* mRNA, maintaining its stability in the cytoplasm, which is closely related to lower tumor differentiation [58]. Moreover, overexpression of SRSFs in patient-derived glioma stem cells induces notable alterations in alternative splicing, promoting the growth and self-renewal of glioma stem cells [59, 60] (Fig. 2).

Elevated expression of SRSF5 and SRSF6 has also been observed in gastrointestinal cancer. Phosphorylation of these proteins by CLK regulates spliceosome assembly, alternative splicing, and gene expression [61–63]. The small molecule inhibitor of CLK, SM08502, penetrates the nucleus of tumor cells and inhibits CLK activity. It suppresses the phosphorylation of SRSF5 and SRSF6 in gastrointestinal cancer, resulting in abnormal splicing of the Wnt signaling pathway and decreased gene expression. Currently, a phase I clinical trial of SM08502 in solid tumors (NCT03355066) is underway to explore its efficacy in tumor treatment [64]. Another kinase regulating phosphorylation of SRSFs, SRPK-1, was also a therapy target of cancers. In non-small cell lung cancer (NSCLC), the chimeric antibody target for SRPK‑1 could significantly suppress growth, migration and invasion of the NSCLC cells [65].

## Functions of SRSFs in normal hematopoiesis

### SRSF2 plays crucial roles in primitive hematopoiesis

**Fig. 3:**
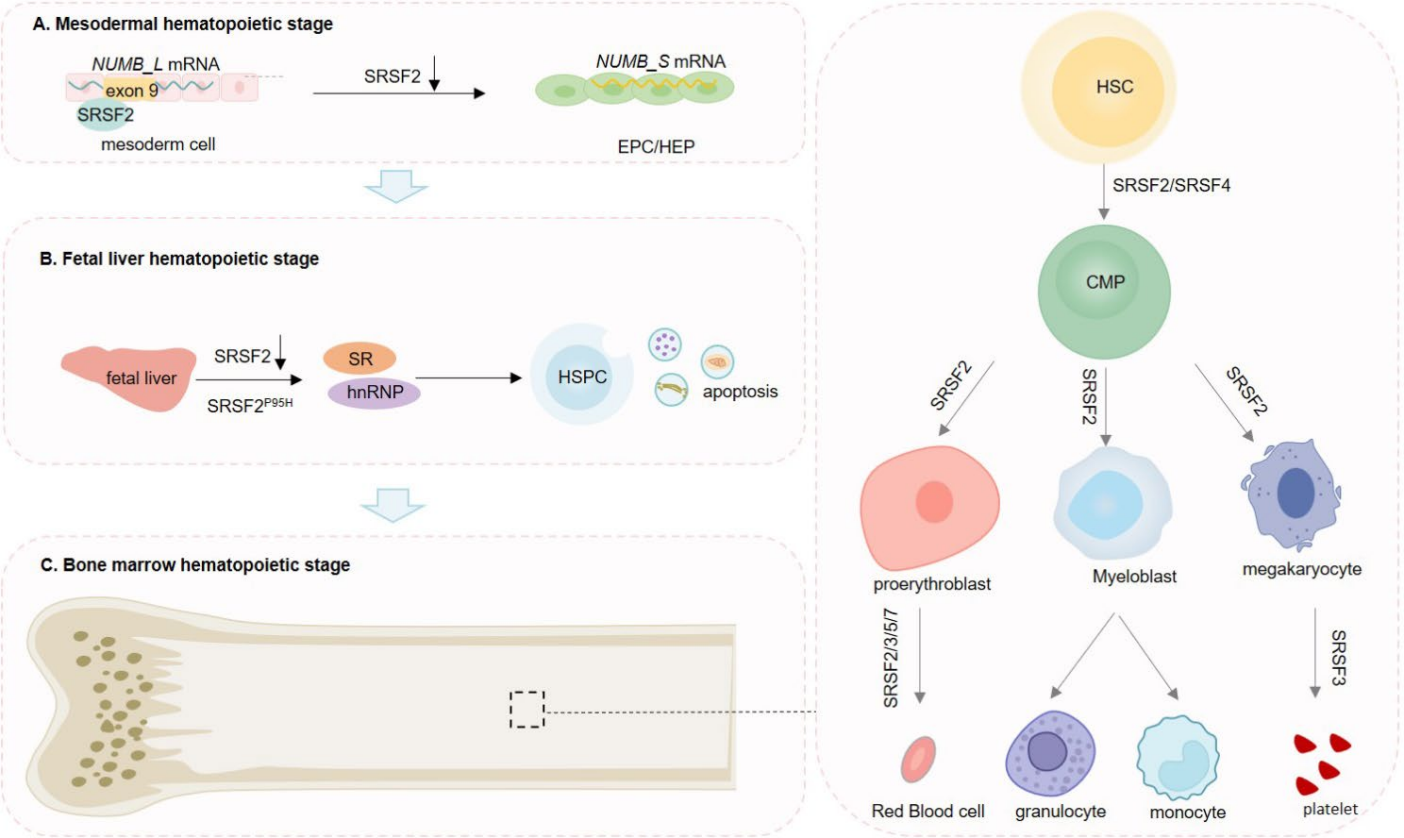
Roles of SRSFs in normal hematopoiesis. (**A**) Mesodermal hematopoietic stage: In mesoderm cells, SRSF2 interacts with *NUMB* exon 9 to regulate the alternative splicing of *NUMB* mRNA and promote the generation of *NUMB_L*. However, the expression level of SRSF2 is down-regulated during EPC generation, resulting in the predominant expression of *NUMB_S* in EPCs. (**B**) Fetal liver hematopoietic stage: fetal hepatocytes lacking Srsf2 exhibit significantly elevated apoptosis levels and reduced hematopoietic stem/progenitor cells (HSPCs) in the liver. SRSF2^P95H^ mutation triggers a series of gene regulatory events that collectively affect hematopoiesis in primary human CD34^ +^ fetal liver cells. For instance, SRSF2^P95H^ targets include several members of the hnRNP and SR families. (**C**) Bone marrow hematopoietic stage: SRSFs play an important role in various stages of adult hematopoiesis

SRSFs play crucial roles in hematopoietic development, and knockdown of *Srsf2* in the hematopoietic system leads to embryonic death [66]. The expression level of SRSF2 changes during the differentiation of mesoderm cells into endothelial progenitor cells (EPCs). Disrupting this process specifically delays the emergence of EPCs and hemogenic endothelial progenitor cells (HEPs) by altering the splicing of *NUMB* mRNA [67]. *NUMB* is essential for normal cell proliferation, and its expression in EPCs peaks during hematopoietic development. However, *NUMB* exhibits various alternative splicing isoforms, primarily distinguished by the length of the phosphotyrosine binding (PTB) domain and proline-rich (PRR) domain. The long isoform, *NUMB_L*, includes exon 9 encoding the 48th amino acid in the PRR domain, while the short isoform, *NUMB_S*, lacks exon 9 [68]. In mesoderm cells, *NUMB_L* is predominantly expressed, while NUMB_S is specifically added in EPCs. NUMB_S plays a vital role in EPC development by activating the NOTCH pathway. SRSF2 interacts with *NUMB* exon 9 to regulate the alternative splicing of *NUMB* mRNA and promote the generation of *NUMB_L*. However, the expression level of SRSF2 is down-regulated during EPC generation, resulting in the predominant expression of *NUMB_S* in EPCs [67] (Fig. 3A).

It has been reported that fetal hepatocytes lacking Srsf2 exhibit significantly elevated apoptosis levels and reduced hematopoietic stem/progenitor cells in the liver [69] (Fig. 3B). The SRSF2^P95H^ mutation targets were related to RNA processing and splicing, which include several members from the hnRNP and SR families, indicating that SRSFs may dominate in RNA splicing [70].

### SRSF2 and SRSF4 can maintain adult hematopoiesis

Knockdown of *Srsf2* in adult mice results in significant reductions in platelet count, bone marrow cell count, and hematopoietic stem cells in bone marrow [69] (Fig. 3C). Various splicing factors, including SRSF2, could be inhibited by telomere dysfunction, leading to altered differentiation in common myeloid progenitor (CMP) and development of classic myelodysplastic syndrome (MDS) phenotype [71].

SRSF2^P95H^ and SRSF2^P95R^ mutations in CD34^+^ hematopoietic stem and progenitor cells (HSPCs) have the potential to trigger a widespread abnormality in alternative splicing. By inducing G2-M phase arrest, these genetic alterations hinder the growth of CD34^ + ^HSPCs, trigger cell apoptosis, and disrupt the process of hematopoietic differentiation towards CMP cells [70, 72]. In particular, SRSF2 gene mutations are identified by an atypical bias in the process of granulo-monocytic differentiation towards monocytes and megakaryo-erythroid differentiation towards megakaryocytes [72].

The protein level of SRSF4 is greatly diminished in Dkc1 hypomorphic mutant mice, which serve as a model for X-linked dyskeratosis congenita (X-DC). X-DC is known for causing bone marrow failure, as well as skin and appendage lesions. LSK cells and CMPs exhibit reduced proliferation capacity due to the decrease in Srsf4 protein expression [73].

### SRSF3 promotes megakaryocyte maturation and platelets production

SRSF3 was reported to be crucial in megakaryocyte maturation and the production of functional platelets (Fig. 3C). Deletion of megakaryocyte-specific *Srsf3* in mice results in megakaryocytosis, characterized by arrested megakaryocyte maturation, abnormal increase in platelet volume, reduced platelet count, impaired platelet function, and aberrant platelet activation in the absence of agonists [5]. There are two main mechanisms through which Srsf3 contributes to these processes. Firstly, Srsf3 regulates the nuclear export of mRNA from megakaryocytes, and its loss leads to the nuclear accumulation of mRNA encoding the cell surface receptors c-MPL and CD41 [5]. Secondly, Srsf3 is involved in the sorting and deposition of megakaryocyte RNA into platelets, as evidenced by significant changes in the RNA repertoire of platelets upon *Srsf3* knockout. For instance, loss of *Srsf3* in megakaryocytes results in decreased expression of *Nbeal2* mRNA in megakaryocytes, whereas its expression is increased in platelets [5].

### Multiple SRSFs participate in the erythroid maturation

The splicing factor SRSF5 plays a critical role in erythroid maturation [74] (Fig. 3C). In early progenitor cells, SRSF5 recognizes the AGACTAG motif in exon 16 of *4.1R* mRNA through its RRM domain, thereby promoting the splicing of *4.1R* mRNA. However, in mature erythrocytes, SRSF5 is hydrolyzed by the proteasome via the RS domain, leading to the retention of exon 16 in *4.1R* mRNA. This allows erythrocytes to synthesize 4.1R protein isoforms with a 10 kDa domain, which are crucial for stabilizing the membrane skeleton.

In addition, the expression level of SRSF2, SRSF3, SRSF6 and SRSF7 also showed significant change of alternative splicing from proerythrocyte to orthochromatic erythroblasts [75] (Fig. 3C).

## SRSFs and hematological malignancies

### Multiple SRSFs are involved in the development of acute myeloid leukemia

The epigenetic alterations in SRSF1 are involved in the progression of acute myeloid leukemia (AML) (Table 1). Firstly, arginine methylation of SRSF1, mediated by protein arginine methyltransferase 5 (PRMT5), promotes the survival of AML cells [76]. Depletion of PRMT5 results in alterations in mRNA alternative splicing and decreased levels of vital proteins that facilitate the growth of AML, including POLD1, POLD2, PPP1R7, PNISR, FDPS, PNKP, and PDCD2 [76]. It was demonstrated that treatment of glioblastoma (GBM) cells with PRMT5 inhibitors compound 5 (CMP5) led to apoptosis of differentiated GBM cells [77]. However, whether CMP5 has the same effect in AML remains to be studied. Secondly, SRSF1 phosphorylation is critical in acute promyelocytic leukemia. It has been shown that retinoic acid promotes the accumulation of protein kinase A in the nucleus, which increases the phosphorylation level of SRSF1. This, in turn, regulates the alternative splicing of the anti-apoptotic factor Mcl-1, induces promyelocytic granulocyte differentiation, and inhibits cell proliferation [78].

**Table 1:** The functional roles of SRSFs in hematologic malignancy

Cancer type	SRSF family members	Role of SRSFs in cancer	References
Acute myeloid leukemia	SRSF1	Arginine methylation of SRSF1 promote the survival of AML cells; phosphorylation level of SRSF1 induces promyelocytic granulocyte differentiation and inhibit cell proliferation.	[76, 78]
	SRSF2	SRSF2 mutation was associated with poor prognosis and shortened survival in primary and secondary AML patients	[79–81]
	SRSF10	SRSF10 promotes the proliferation of AML cells	[82]
Myelodysplastic syndromes	SRSF2	MDS patients with SRSF2 mutation had an inferior overall survival	[83–85]
ALL	SRSF1	The expression level of SRSF1 were significantly up-regulated in ALL; phosphorylation of SRSF1 at tyrosine 19 enhances cell proliferation	[86, 87]
T-ALL	SRSF6	The high expression of SRSF6 in T-ALL was associated with poor prognosis	[46]
B-ALL	SRSF3	The expression level of SRSF3 was lower in relapsed B-ALL, leading to exon 2 skipping of CD19 and expression of the N-terminally truncated CD19 variant in B-ALL cells, which fails to trigger killing by CART-19	[88]
Myeloproliferative neoplasm	SRSF2	SRSF2 mutation can promote the progression of primary myelofibrosis and leukemic transformation	[89–91]
Chronic myeloid leukemia	SRSF1	High SRSF1 levels in the bone marrow of CML patients at presentation correlated with poorer responses to tyrosine kinase inhibitors	[92]
Chronic myelomonocytic leukemia	SRSF2	Whether SRSF2 mutation was associated with prognosis in CMML patients is controversial	[93, 94]
Clonal hematopoiesis	SRSF2	SRSF2 mutation was associated with malignant transformation to myeloid malignancies	[95]

**Fig. 4:**
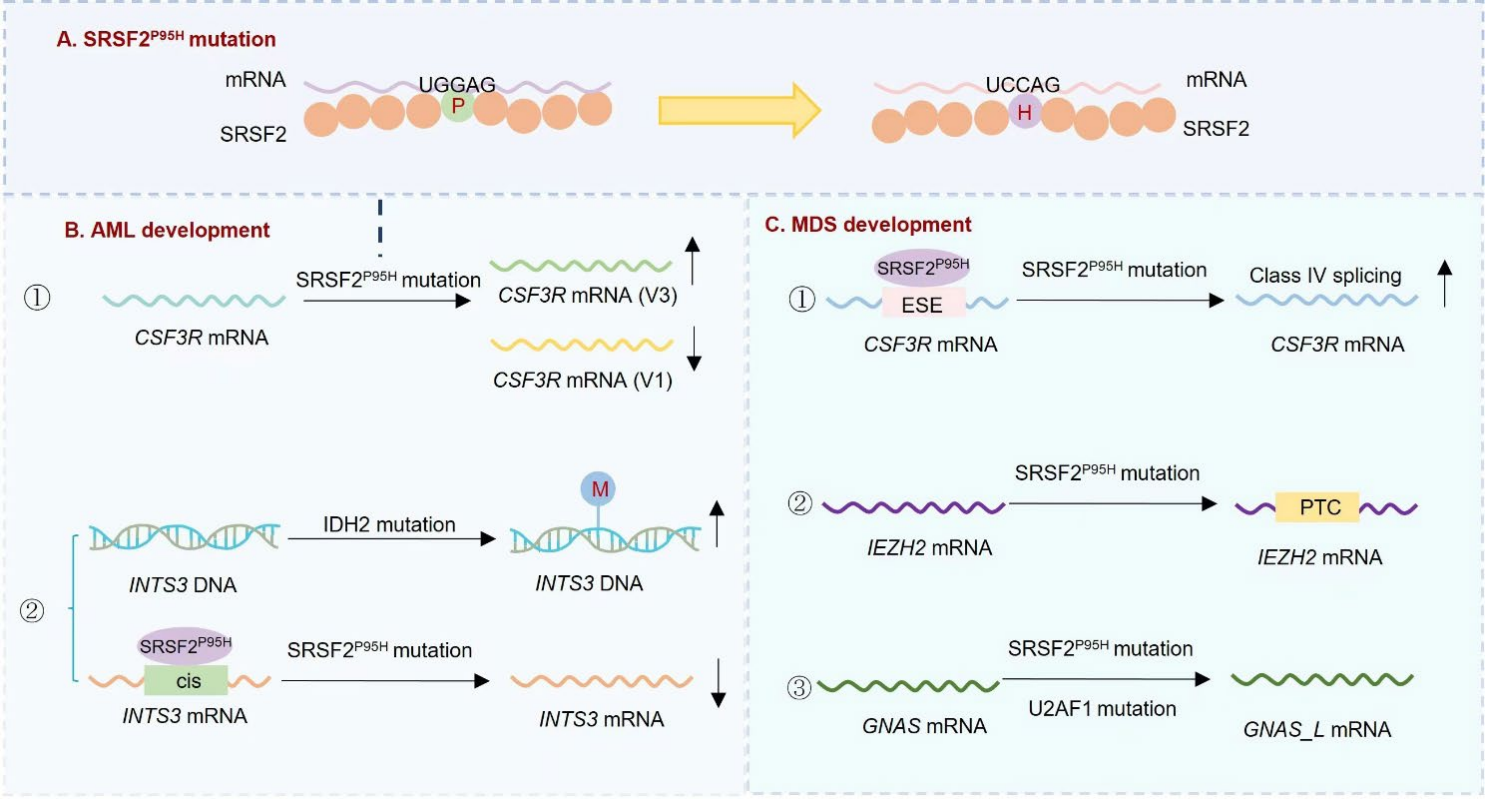
Roles of SRSF2^P95H^ mutation in AML and MDS. (**A**) The proline-to-histidine mutation at position 95 of SRSF2 (SRSF2^P95H^) alters the spatial structure of SRSF2, affecting its recognition of splice sites. The SRSF2^P95H^ mutation binds more tightly to RNA sites containing UCCAG but binds less tightly to UGGAG sites compared to the wild type. (**B**) In AML, SRSF2 mutations affect the splicing of *CSF3R* mRNA, leading to the significant increase in the *V3/V1*. In addition, co-mutations of SRSF2 with IDH2 can synergistically promote the development of AML. Mutant IDH2 increase DNA methylation of *INTS3*, and mutant SRSF2 binds to cis elements in *INTS3* mRNA to change the splicing, which lead to reduced expression of INTS3 and contribute to leukemogenesis. (**C**) SRSF2^P95H^ promotes Class IV splicing by binding to ESE sequences in *CSF3R* exon 17, leading to dysgranulopoiesis. In addition, SRSF2 mutation can cause *EZH2* mRNA isoforms to contain a premature termination codon (PTC) degraded by NMD pathway, resulting in impaired erythrogenesis and increased ineffective hematopoiesis. Co-mutation of SRSF2 with splicing factor U2AF1 cause mis-splicing of the long GNAS isoform (GNAS_L) mRNA

Approximately 25% of AML patients exhibit SRSF2 mutations, which are linked to unfavorable prognosis and reduced survival rates in both primary and secondary AML patients [79–81] (Table 1). The proline-to-histidine mutation at position 95 of SRSF2 (SRSF2^P95H^) alters the spatial structure of SRSF2, affecting its recognition of splice sites. This mutation leads to the mis-regulation of 548 splicing events, with 374 involving the inclusion of cassette exons (Fig. 4A). The UCCA/UG and UGGA/UG motifs are enriched in the more-included and more-excluded exons, respectively. The SRSF2^P95H^ mutation has a stronger affinity for RNA sites that have UCCAG, and its affinity for UGGAG sites is weaker compared to the wild type, resulting in a higher frequency of exon inclusion [96]. For instance, SRSF2 mutations affect the splicing of *CSF3R* mRNA [97]. CSF3R has two splice variants, V3 and V1. The presence of V3 alone confers a hypo-proliferative characteristic and impaired JAK-STAT activation in hematopoietic cells. AML patients with SRSF2 mutations exhibited a notable rise in the V3/V1 ratio. The V3/V1 ratio was reduced by the knockout of SRSF2, indicating that CSF3R is affected by SRSF2 mutations [98] (Fig. 4B). Targeting the changes in alternative splicing caused by SRSF2 mutations may be a therapeutic approach for AML. The spliceosome inhibitor E7107 treatment could significantly reduce leukemic burden, and AML with SRSF2^P95H^ mutations is preferentially susceptible to spliceosome inhibitor, unlike leukemias that do not have these mutations [99].

Co-mutation of SRSF2 with other genes can synergistically promote the development of AML [100]. The concurrence of genetic changes in IDH2 and SRSF2 among individuals with AML results in synchronized impacts on the epigenome and RNA splicing, which play a role in the development of leukemia. AML cells with mutations in both IDH2 and SRSF2 display abnormal splicing and decreased levels of INTS3, a component of the integrator complex. This leads to enhanced RNA polymerase II (RNAPII) stalling. The abnormal splicing of INTS3 caused by the mutant SRSF2 binding to cis elements in *INTS3* mRNA, along with the increased DNA methylation of INTS3 caused by the mutant IDH2, plays a role in the development of leukemia [100] (Fig. 4B).

Zhong et al. analyzed the expression characteristics of SRSFs in AML samples from the TCGA database and found that SRSF4 and SRSF9 were significantly down-regulated, while SRSF8, SRSF5, SRSF2, SRSF7, SRSF10, and SRSF12 were up-regulated compared to normal samples. They also confirmed that SRSF10 expression was significantly up-regulated in clinical AML samples. Knockdown of SRSF10 inhibited AML cell proliferation, promoted apoptosis, and induced G1 phase arrest in the cell cycle [82] (Table 1).

### SRSF2^P95H^ mutation plays the carcinogenic role in myelodysplastic syndromes

In myelodysplastic syndromes (MDS), oncogenic mutations of the splicing factor SRSF2^P95H^ are common [101–103]. The presence of SRSF2 mutation showed a strong correlation with being male and having advanced age. Patients with SRSF2 mutation had a lower overall survival rate [83–85] (Table 1). It was demonstrated that Srsf2-mutated HSPCs exhibited a series of aberrant splicing [104, 105]. The presence of SRSF2 mutation led to a decrease in HSPCs and resulted in abnormalities in their differentiation. Additionally, it facilitated the conversion of hematopoietic stem cells into myeloid tumors, which were characterized by anemia, leukopenia, and erythroid dysplasia [105–107]. For example, SRSF2^P95H^ enhances the process of Class IV splicing by attaching to ESE patterns in exon 17 of *CSF3R* mRNA, leading to dysgranulopoiesis [108] (Fig. 4C). In addition, SRSF2 mutation can cause *EZH2* mRNA isoforms to contain a premature termination codon (PTC) degraded by NMD pathway, resulting in impaired erythrogenesis and increased ineffective hematopoiesis [103, 109]. SRSF2^P95H^ mutation could also cause elevated levels of DNA: RNA intermediates (R-loops) and ATR pathway activation in MDS CD34^+^ cells [110, 111].

Co-mutation of SRSF2 and other important genes, such as U2AF1 and RUNX1, was common in MDS. Mutant U2AF1 and SRSF2 can cause convergent mis-splicing of the GNAS isoform, leading to the activation of G protein and ERK/MAPK signaling. This activation drives MDS and makes mutant cells responsive to MEK inhibition [112]. Huang et al. constructed a mouse model by combining the Runx1 knockout with the Srsf2^P95H^ mutation, resulting in multilineage hematopoietic defects. This was accompanied by mis-splicing of genes that are specifically abundant in the DNA damage response and cell cycle checkpoint pathways [113] (Fig. 4C).

It was suggested that the pathways of cell-cycle and DNA damage response are necessary for the survival of Srsf2^P95H/+^ cells. Additionally, it was proposed that palbociclib, an inhibitor of cyclin dependent kinase 6 (CDK6), could serve as an alternative therapeutic choice for targeting cancers with SRSF2 mutations [114].

### High expression of SRSF1 and SRSF6 are associated with poor prognosis in T-cell acute lymphoblastic leukemia

In acute lymphoblastic leukemia (ALL), the splicing profiles of T-ALL patients were significantly different from those of normal T cells. In ALL patients, the level of SRSF1 expression was notably increased, while the expression of SRSF1 returned to normal after complete remission with cytosine arabinosine or vincristine, but increased again in relapsed samples [86] (Table 1). Knockdown of SRSF1 resulted in increased apoptosis and inhibited the proliferation of ALL cells [86]. The phosphorylation of SRSF1 at Tyr-19 was detected in newly diagnosed ALL samples, but it was not found in samples from complete remission or normal controls. Phosphorylation of SRSF1 at tyrosine 19 promotes cell proliferation and enhances the ability of colony-forming units [87]. The subcellular localization of SRSF1 was impacted by missense mutants that altered Tyr-19 phosphorylation in contrast to SRSF1 wild-type cells.

High expression of SRSF6 in T-ALL was linked to poor prognosis, and the ubiquitin-specific peptidase 7 (USP7) could improve the protein level of SRSF6 through active de-ubiquitination and promote the proliferation of T-ALL cells [46] (Table 1). The small molecule H3B-8800, which acts as a splicing inhibitor by binding to Splicing Factor 3B1 (SF3B1), can affect the splicing of proteasome transcripts and proteasome activity and synergize with proteasome inhibitors to inhibit the growth of T-ALL [115]. It was reported that the splicing factors SRSF5 and SRSF6 can also cause a change in the splicing of *CCAR1* mRNA, shifting it from the complete version to a shorter variant, and maintain the growth of cancer cells [116].

### Low expression of SRSF3 causes resistance to CART-19 in recurrent B-cell neoplasms

CD19 is an important therapy target for CART in B-ALL [117]. Splicing factor SRSF3 was associated with retention of exon 2 in *CD19* mRNA. However, the levels of SRSF3 were reduced in relapsed B-ALL, resulting in the exclusion of exon 2 and the emergence of a truncated form of CD19 in B-ALL cells. This truncated variant of CD19 is unable to activate the killing response by CART-19 [88] (Table 1). Thus, enhancing the expression of SRSF3 may enhance the survival rate of individuals experiencing recurrent B-cell neoplasms.

### SRSF2 mutations are linked to poor prognosis of myeloproliferative neoplasm

Myeloproliferative neoplasms (MPN) include primary myelofibrosis (PMF), polycythemia vera (PV) and essential thrombocythemia (ET), which are morphologically and molecularly similar [118]. The frequency of *SRSF2* mutation in PMF patients was about 17%, mainly occurring in residue P95, which is an independent marker of poor prognosis in PMF patients [89–91, 119] (Table 1). Vallapureddy et al. collected and analyzed the clinical and cytogenetical characteristics of 1306 PMF patients and found that SRSF2 mutations were predictors of leukemic transformation in the first 5 years of diagnosis [89]. Lasho et al. screened 187 PMF patients and confirmed significant associations between SRSF2 mutations and older age, IDH mutations, and elevated DIPSS-plus risk classification. Mutations in SRSF2 were linked to reduced overall and leukemia-free survival [119]. In addition, it was demonstrated that SRSF2 mutations was associated with poorer overall in PV [120]. SRSF2 mutations can also promote rapid blast transformation of MPN [121, 122].

### SRSF1 mediates imatinib resistance in chronic myeloid leukemia

SRSF1 expression was found to be elevated in primary CD34^+^ progenitors of chronic phase chronic myeloid leukemia (CML) compared to the normal progenitors, which was mediated by BCR-ABL1 and cytokine. Elevated levels of SRSF1 upregulated PRKCH and PLCH1 mechanistically, leading to impaired imatinib sensitivity in patients with CML [92] (Table 1).

However, in East Asian, the potential mechanism of imatinib resistance mediated by SRSF1 was different. The deletion polymorphism located in intron 2 of the *BIM* mRNA caused the splicing of *BIM* mRNA to shift from exon 4 to exon 3 mediated by SRSF1, leading to the production of splice isoforms that do not contain the pro-apoptotic BH3 domain encoded by exon 4 [123]. The removal of exon 4 in *BIM* mRNA splicing hinders the ability of TKIs to trigger apoptosis [123]. It was suggested that SRSF1 could be a potential target for treating imatinib-resistant CML caused by the deletion polymorphism in BIM.

### The controversial roles of SRSF2 mutations in chronic myelomonocytic leukemia

Patnaik et al. analyzed the cytogenetics of 226 patients with chronic myelomonocytic leukemia (CMML) and found that SRSF2 mutation is the most commonly mutated gene in the spliceosome with a mutational frequency of 40% (all affecting P95). And it is not associated with prognosis of CMML patients [93]. However, Yuan et al. reported that CMML patients with SRSF2 exhibited reduced overall survival (OS) and progression-free survival (PFS) [94] (Table 1). In CMML patients with mutations in the splicing factor SRSF2, there were multiple abnormal splicing occurrences observed in genes related to DNA repair [124]. Furthermore, a portion of the Srsf2/Tet2 mutants exhibit an elevated proliferation of granulocytes and a unique proliferation of monocytes (myelomonocytic hyperplasia), characterized by an increase in immature promonocytes and monoblasts, as well as binucleate promonocytes [125].

### SRSF2 mutations plan an important role in clonal hematopoiesis

Clonal expansions driven by somatic mutations in the hematopoietic system was defined clonal hematopoiesis [126]. Fabre et al. found that SRSF2 mutations was one of the common clonal hematopoiesis-associated mutations. The mutation exhibiting unique behavior was SRSF2^P95H^, which was linked to notably quicker expansion in contrast to other SRSF2 mutations. Clonal expansion triggered by SRSF2^P95H^ mutation typically occurred after the age of 30 and expanded 50% per year, far more than DNMT3A and TP53 mutations (5%) [127]. In addition, the risk of leukemogenesis was higher in patients with SRSF2^P95H^ mutation than those with DNMT3A mutations. Recurrent microhomology causes the decreased copy number of SRSF2 in early pluripotent HSCs, which is also closely related to the development of clonal hematopoiesis [128]. Moreover, SRSF2 mutations was associated with malignant transformation to myeloid malignancies of chronic idiopathic neutropenia patients [95] (Table 1).

## Conclusions and open questions

In conclusion, the review has introduced the basic functions of SRSFs in hematopoietic development and malignancies. However, there are still some problems waiting to be solved. First, it has been demonstrated that SRSFs could regulate various forms of alternative splicing, but what determines the specific splicing form is unclear. Second, although SRSF2^P95H^ mutations are common in a variety of hematological malignancies, including AML, MDS, MPN, CMML, and clonal hematopoiesis, the therapeutic strategies targeting this mutation remain to be further studied. Moreover, the specific inhibitors of SRSFs are less developed. Currently, only a kind of small molecule inhibitor of CLK, which can suppress the phosphorylation of SRSF5 and SRSF6, have been applied in a phase I clinical trial of solid tumors, and the clinical efficacy is needed to be further explored. Therefore, more specific inhibitors of SRSFs need to be developed to better serve patients with hematologic malignancies and other solid tumors and improve the prognosis of patients.

## Data Availability

Not applicable.

## Abbreviations

SRSFs: Serine/arginine-rich splicing factors
pre-RNA: precursor RNA
snRNPs: small nuclear ribonucleoprotein particles
SREs: Splicing regulatory elements
ESEs: Exonic splicing enhancers
ESSs: Exonic splicing silencers
ISEs: Intronic splicing enhancers
ISSs: Intronic splicing silencers
RRMs: RNA recognition domains
RS: Serine/arginine-rich domain
5'ss: 5’ splice sites
3'ss: 3’ splice sites
BPSs: Branch point sites
SF1: Splicing factor 1
CDS: Coding sequence
UTR: Untranslated region
PTC: Premature termination codon
NMD: Nonsense-mediated mRNA decay
TDP43: TAR DNA-binding protein-43
P-TEFb: Positive transcription elongation factor b
PDCD4: Programmed cell death 4
CLK: CDC like kinase
SRPK: SR protein kinases
NSCLC: Non-small cell lung cancer
EPCs: Endothelial progenitor cells
HEPs: Hemogenic endothelial progenitor cells
CMP: Common myeloid progenitor
MDS: Myelodysplastic syndrome
HSPCs: Hematopoietic stem and progenitor cells
AML: Acute myeloid leukemia
PRMT5: Protein arginine methyltransferase 5
GBM: Glioblastoma
SRSF2^P95H^: Mutation at position 95 of SRSF2
ALL: Acute lymphoblastic leukemia
USP7: Ubiquitin-specific peptidase 7
MPN: Myeloproliferative neoplasms
PMF: Primary myelofibrosis
PV: Polycythemia vera
ET: Essential thrombocythemia
CML: Chronic myeloid leukemia
CMML: Chronic myelomonocytic leukemia
OS: Overall survival
PFS: Progression-free survival
